# Understanding the Relationship Between Early Elementary Children’s ADHD Symptoms and Teachers’ Needs Supportive Practices

**DOI:** 10.1177/10870547261427102

**Published:** 2026-04-15

**Authors:** Melissa Kang, Anne-Claude V. Bédard, Angela Pyle, Frédéric Guay, Rhonda Martinussen, André Plamondon

**Affiliations:** 1 University of Toronto, ON, Canada; 2 Université Laval, Quebec City, QC, Canada

**Keywords:** ADHD symptoms, early elementary, academic motivation, student engagement, needs supportive practices

## Abstract

**Objective:**

Many children with diagnosed and subclinical ADHD struggle with low academic motivation in early elementary. Fortunately, teachers’ needs’ supportive practices (NSPs) can mitigate motivational challenges and protect against disengagement and underachievement. Teachers’ NSPs include autonomy support, structure, and positive student-teacher relationships that target children’s motivational resources (i.e., autonomy, competency, and relatedness) needed to engage with school materials. Particularly, autonomy support practices fuel children’s feelings of autonomy, structure caters to feelings of competency, and positive student-teacher relationships provide feelings of belonging. Yet, it is unknown how ADHD symptoms in early elementary are associated with teachers’ NSPs.

**Methods:**

One hundred and fifty-four first-grade students and 25 teachers from three school boards participated. We assessed children’s perception of their teachers’ autonomy support and structure and their standardised achievement. We also assessed teachers’ perceptions of students’ ADHD symptoms, student-teacher relationship quality, and conduct problems. Three linear regression analyses were performed with ADHD symptoms as the predictor and NSPs as the dependent variable. Sex, conduct problems, and achievement scores were included as covariates.

**Results:**

Sex (i.e., males), greater teacher-reported ADHD symptoms, and more teacher-reported conduct problems were associated with worse teacher-reported student-teacher relationship quality. Teacher-reported ADHD symptoms were positively related to student-reported autonomy support, while teacher-reported conduct problems were negatively associated with student-reported autonomy support. Teacher-reported ADHD symptoms and key covariates did not predict student-reported structure.

**Conclusion:**

Our study illustrates the need to further evaluate how best to support teachers managing disruptive behaviours in early elementary to protect the motivational needs of young children with ADHD symptoms.

## Public Significance Statement

Many young children with ADHD symptoms struggle to feel motivated in school. Our study showed that the behavioural problems associated with ADHD symptoms likely negatively affect teachers’ impressions of these students, which can undermine children’s motivation and desire to engage in learning. This highlights the need to examine best practices in managing early elementary children who display challenging behaviours that interfere with the classroom, consequently contributing to worse student-teacher relationship quality and controlling instructional practices.

## ADHD Symptoms in the Classroom and Academic Motivation

Children diagnosed with ADHD or those who exhibit symptoms associated with this disorder often face motivational impairments in the classroom that worsen over time (Rogers et al., 2015; Volpe et al., 2006). These motivation impairments can, in turn, predict future underachievement and poor school adjustment independent of prior achievement and interpersonal skills (Berchiatti et al., 2021; Volpe et al., 2006). Negative associations between ADHD symptoms and academic motivation are observed early in development when children begin first grade (Ogg et al., 2016; Rogers et al., 2015). Thus, identifying the contextual factors underpinning the motivational difficulties of children with ADHD symptoms is critical, especially when they begin elementary school. Addressing these factors early on can help minimise negative impacts on their academic and socio-emotional functioning in the long run.

### Self-Determination Theory

Self-Determination Theory suggests that humans are innately driven to learn, grow, and connect with others (Deci & Ryan, 1985, 2000). Specifically, these innate tendencies are facilitated by the satisfaction of three basic psychological needs: *autonomy*, or one’s desire to experience choice and control over their learning; *competence*, to feel skilful and efficacious when performing school tasks; and *relatedness*, to feel a sense of belonging with significant others (Deci & Ryan, 1985, 2000, 2020). These needs can be met through needs’ supportive practices (NSP) provided by people in authority positions, such as parents or teachers, and in more egalitarian relationships, such as the one with peers. In this study, the focus is on teachers’ NSPs, which include *autonomy support*, provision of *structure*, and *relatedness.* The latter refers to positive *student-teacher relationships* in the school context (Connell & Wellborn, 1991). According to Furrer et al. (2008), Teachers’ NSPs provide children with a classroom environment that promotes a drive to learn and include instructional support that emphasises choice, competence, and caring.

Children who experience high teacher provisions of NSPs are likely to feel autonomously motivated (i.e., interest toward school and valuing school) and express their motivation through enhanced engagement in learning (Bureau et al., 2022; Oga-Baldwin & Nakata, 2015; Van den Berghe et al., 2015). While teachers’ instructional styles vary from class to class, greater differences are observed in how teachers cater NSPs to individual students (Oga-Baldwin & Nakata, 2015). In other words, children in the same classroom can harbour very different perceptions of the same teacher and their NSPs (Oga-Baldwin & Nakata, 2015; Skinner et al., 2009; Van den Berghe et al., 2015). Consequently, teachers and children may differ in their perception of the provision of NSPs, with children’s perceptions of their teachers more closely related to their self-reports of their motivation and engagement levels (Oga-Baldwin & Nakata, 2015; Van den Berghe et al., 2015). Thus, it is essential to consider children’s perspectives to predict better outcomes (Skinner et al., 2009; Van den Berghe et al., 2015).

Many children with elevated ADHD symptoms are less likely to be academically motivated than their typically developing peers (Portilla et al., 2014; Volpe et al., 2006). From the perspective of Self-Determination Theory, their motivational deficits may be tied to having unmet psychological needs through inadequate provisions of NSPs (Connell & Wellborn, 1991; Deci & Ryan, 1985). Prior research has either encountered mixed findings (e.g., autonomy support and structure, see Ogg et al., 2016; Sanders, 2015) or focussed predominantly on older, typically developing students (e.g., student-teacher relationships, see Rogers et al., 2015). Examining these relationships will provide deeper insight into how academic motivation develops and how it relates to emotional and academic outcomes in early elementary classrooms.

## Considering Needs Supportive Practices Among Children With ADHD Symptoms

### ADHD and Student-Teacher Relationships

Among the different teacher NSPs, researchers have chiefly explored the negative student-teacher relationship quality among children with diagnosed and subclinical ADHD (Ewe, 2019; Zendarski et al., 2020). These children often encounter difficulties engaging with classroom materials and exercising appropriate control over their thoughts, feelings, and behaviours (Portilla et al., 2014; Rushton et al., 2020; Vile Junod et al., 2006). Teachers’ observations of and interactions with these classroom behaviours contribute to their negative impression of the child, as evidenced by teachers’ reports of experiencing more conflict, greater emotional stress, and less emotional closeness with students with ADHD symptoms than with children having a more typical development (DeShazer et al., 2023; Ewe, 2019; Greene et al., 2002; Hamre & Pianta, 2001; Portilla et al., 2014). In addition to ADHD symptoms, poor student-teacher relationship quality has been associated with being a male, conduct problems, and underachievement (Crum et al., 2015; Jerome et al., 2009; Zendarski et al., 2020).

Furthermore, there is a specific need to understand the student-teacher relationship quality for children with ADHD symptoms transitioning from kindergarten (Zendarski et al., 2020). First grade classrooms shift from play-based learning to a traditional teacher-directed approach with increased academic independence and time spent seated (Portilla et al., 2014; Zendarski et al., 2020). This environmental shift likely challenges children’s self-regulatory abilities and affects how children and teachers interact emotionally with one another (Guay et al., 2019; Vile Junod et al., 2006; Zendarski et al., 2020). Unfortunately, few studies have explicitly evaluated how children’s ADHD symptoms may account for their student-teacher relationship quality during this critical transition into elementary school (Portilla et al., 2014; Zendarski et al., 2020). For instance, prior studies assessed the relationship between children’s ADHD symptoms and their student-relationship quality within a broad age range (e.g., Portilla et al., 2014, ages 4–6 years; Zendarski et al., 2020, ages 6–8 years), which misses the key period when young students adjust to structured learning. Our study examines this association exclusively among first-grade students, which may be important for early intervention (Berchiatti et al., 2021; Volpe et al., 2006).

### ADHD and Autonomy Support

Autonomy support has been studied extensively with typically developing children and youth (Bureau et al., 2022; Reeve et al., 2002; Reeve & Jang, 2006), however few studies have examined the relationship between autonomy support and ADHD symptoms in early elementary classrooms (Rogers & Tannock, 2018). Rogers and Tannock (2018) found that children ages 5 to 11 years with ADHD symptoms tended to perceive their teachers as more controlling than those without ADHD symptoms, independent of coexisting behaviour problems and achievement scores. The partial eta-squared effect size from this preliminary finding was relatively modest (η^2^ = .10), suggesting that children with ADHD symptoms across elementary classrooms may have unmet autonomy needs (Rogers & Tannock, 2018). Other studies from the parenting styles literature suggest that challenging child behaviours (i.e., hyperactivity/impulsivity) tend to elicit less parental autonomy support and prompt greater intrusiveness and verbal/physical punishment (i.e., *harsh control*) from parents (Hoeve et al., 2009; Pinquart, 2017). The dynamic experienced by parents and children with disruptive behaviours may mirror that experienced by early elementary teachers and young children with ADHD symptoms. The symptoms of ADHD in children may trigger negative emotions in teachers, such as frustration and panic. These emotions, in turn, may lead to increased harsh control and reduced use of autonomy-supportive practices, such as offering choices to students, using supportive language, and acknowledging the child’s negative emotions and individual perspectives (Hoeve et al., 2009; Pinquart, 2017).

Among the instructional approaches that support children’s transition from kindergarten to first grade are teachers’ autonomy support practices, which are critical in helping to augment or undermine children’s feelings of choice and control in their learning (Niemiec & Ryan, 2009). While previous studies have pooled students across elementary grades (Rogers & Tannock, 2018; Volpe et al., 2006), no studies have examined exclusively first grade children’s perception of teachers’ autonomy support, especially among those presenting with ADHD symptoms. Focussing on this limited period and incorporating children’s voices is necessary to understand how and when early elementary children’s ADHD symptoms are associated with worse academic motivation (Rogers et al., 2015; Vile Junod et al., 2006; Volpe et al., 2006).

### ADHD and Structure

In Roger and Tannock’s (2018) study, children exhibiting heightened ADHD symptoms expressed lower levels of competence compared to their non-ADHD peers, regardless of their academic proficiency and pre-existing behaviour problems. According to Self-Determination Theory, children’s feelings of competence are likely associated with structure (i.e., teacher provisions of explicit rules, guidelines, and expectations in the classroom) encountered in the classroom (Ryan & Deci, 2020). Given that structure is related to students’ feelings of competency (Guay et al., 2017; Rogers & Tannock, 2018), this NSP may be vital in supporting students with ADHD and ADHD symptoms who are struggling with feeling less competent and ineffective in academic tasks (Rogers & Tannock, 2018). However, like teachers’ autonomy support, there is limited research on the relationship between ADHD symptoms and teachers’ structuring within early elementary classrooms. For example, Loopers et al. (2023)’s study focussed on older students in examining differences between students with and without special needs receiving teachers’ NSPs.

Given this lack of research, we looked to parenting styles studies on *behavioural control.* Parental behavioural control, like teachers’ structure practices in the classroom, involves communicating clear and consistent expectations and monitoring a child’s behaviours relative to these expectations (Barber et al., 2005). Meta-analyses point to greater problem behaviours predicting fewer parental behavioural control strategies (Hoeve et al., 2009; Pinquart, 2017). This general trend can be explained by the theory of coercive family dynamics, which postulates that children’s problem behaviours and negative parenting practices exacerbate each other (Pinquart, 2017; Smith et al., 2014). Again, this parent-child relational dynamic may parallel student-teacher interactions in classroom settings. In particular, when a child showcases challenging, disruptive behaviours that signal disengagement or noncompliance with teacher instructions, their behaviours may be met with adverse emotional reactions and inadequate structuring from teachers that decrease chances for children with ADHD symptoms to feel competent (Dale et al., 2022; Pinquart, 2017).

Other relevant research avenues focus on the self-regulatory and motivational deficits of children with ADHD and ADHD symptoms. Namely, children with diagnosed and ADHD symptoms tend to struggle with planning, organising, and monitoring their learning behaviours and rely on extrinsic rewards (e.g., grades) to feel academically motivated (Luman et al., 2005; Morsink et al., 2021; Reddy et al., 2018). When there is less structure in the classroom, children with ADHD symptoms are more likely to suffer compared to those without ADHD symptoms as a function of their cognitive impairments in self-regulation and motivation (Luman et al., 2005; Morsink et al., 2021; Reddy et al., 2018).

During the transition to first grade, children experience a change in the learning environment (e.g., less play-based learning and increased seated tasks) that likely requires teachers to shift their instructional approaches (Sanders, 2015). Unfortunately, no study has examined first grade children’s perceptions of teachers’ structure and its association with ADHD symptoms. Examining this relationship will allow us to better understand the developmental trends in feelings of competency and connected emotional and academic outcomes in early elementary classrooms (Rogers et al., 2015; Vile Junod et al., 2006; Volpe et al., 2006).

## Current Study

The current study aimed to understand the relationships between first-grade children’s ADHD symptoms and their teachers’ NSPs. We hypothesised that greater teacher-reported ADHD symptoms would predict worse teacher-reported student-teacher relationships (Ewe, 2019; Zendarski et al., 2020), fewer student-reported autonomy support practices (Pinquart, 2017), and less student-reported structure in first grade students (Guay et al., 2017). Moreover, we expected that all these relationships would hold when considering sex (i.e., males), conduct problems, and academic competency (measured by reading ability), as suggested by prior research (Crum et al., 2015; Jerome et al., 2009; Rogers et al., 2015; Zendarski et al., 2020).

## Methods

### Recruitment and Participants

One hundred and fifty-four first grade students and 25 first-grade teachers participated in the study. This study was part of a larger project examining the pathways of inattentive behaviours on student engagement and motivation in early elementary school (southern Ontario, Canada). Teachers and their students were recruited from three school boards. First, the principals of different schools consented to participate in this study. Teachers then consented to participate and sent forms to their students’ parents. Consenting parents were sent demographic questionnaires, but only 34% of parent packages were returned, resulting in limited demographic information for each student (i.e., children’s academic supports, parental education level, and language spoken at home; see Table 1). Due to the COVID-19 pandemic in the spring of 2020, data collection was disrupted and many teachers and parents encountered difficulty completing and returning demographic questionnaires. The university and school boards’ ethics committees stipulated maximum participation of up to 10 Grade 1 teachers and eight students per class within each school until a maximum of 40 students per board could be recruited for the entire school year (School Board 1 = 48, School Board 2 = 51, and School Board 3 = 55).

**Table 1.** Sample Demographic Characteristics for Teachers and Students.

Teacher characteristics	*n*	%
Informant type		
Male	1	4
Female	24	96
Age		
20–30	1	4
30–40	6	24
40–50	3	12
50+	4	16
No response	11	44
Years teaching		
1–10	4	16
11–19	4	16
20+	6	24
No response	11	44
Years teaching grade 1		
1–10	12	48
11–19	1	4
20+	1	4
No response	11	44
Educational level		
Undergraduate	10	40
Master’s	4	16
Doctorate	0	0
No response	11	44
Special education qualification		
Yes	8	32
No	5	20
No response	12	48
Student characteristics	*n*	%
Sex		
Female	76	49
Male	79	51
Student status^a^		
Special needs	—	7–26
Language other than English	—	2–48
Born outside of the country	—	<1–24
At/above provincial academic expectations^a^	—	71
Reading	—	60–96
Writing	—	57–96
Mathematics	—	51–92

*Note*. The table outlines the data from 14 teachers who completed the demographics survey and the 154 Grade 1 children who participated.

a Data supplemented using the 2017 to 2018 Education and Quality Accountability Office (EQAO). Data from children at the present study’s schools in Grade 3.

### Procedure

Data collection occurred over 3 years (2017–2019) in the spring term of the school year. The timing of the data collection ensured that teachers and children benefited from observing one another’s practices and behaviours throughout the school year. Three cohorts of students were recruited in the spring terms, with 22 students recruited in cohort 1, 44 in cohort 2, and 88 in cohort 3. Twenty-eight classrooms participated in the study from 14 schools with 25 classroom teachers (three teachers participated in more than one cohort). Teachers completed rating scales on one to eight students per classroom, in accordance with school board ethics regulations. Due to limited demographic information, we supplemented the missing data with provincial data collected from 2017 to 2018, reflecting student characteristics in Grade 3 (see Table 1), to help illustrate the general characteristics of the student sample. The province’s Education Quality and Accountability Office (EQAO) annually assess Grade 3 students’ academic abilities and makes the information publicly available. Among the schools evaluated, the children born outside of Canada ranged from less than 1% to 14%, and the proportion of students with a primary language other than English ranged from 2% to 28%. In addition, 7% to 26% of students identified as those with special education needs (excluding giftedness).

Child participants completed a battery of questionnaires that assessed their perception of their teachers’ autonomy support and structure in the classroom. A trained examiner administered all child questionnaires individually on a laptop through the programme Inquisit (*Millisecond*, 2016). The examiner read aloud each item, and the child chose their response, which the examiner selected on the screen. As well, children’s understanding of rating scales was assessed using primer items (i.e., “I like to go to parties” and “Blue is my favourite colour”). This priming method has increased the reliability of young children’s responses to questions with Likert scales (Marsh et al., 2008). Children also completed a standardised reading achievement assessment to assess their academic competency. Finally, teachers completed questionnaires on their perceptions of their students’ ADHD symptoms, student-teacher relationship quality, and conduct problems.

### Measures

#### ADHD Symptoms

Teachers completed the Strengths and Weaknesses of ADHD-symptoms and Normal Behaviour (SWAN rating scale; Swanson et al., 2012) to assess variation in child participant inattention and hyperactivity/impulsivity symptoms (Arnett et al., 2013). The 18-item questionnaire (α = .99) asked teachers to report children’s symptoms on a 7-point Likert scale from 3 (reflecting high ADHD traits) to −3 (low ADHD traits). The scale’s internal consistency within community samples was high, with an a of .94 to .98 (Plamondon & Martinussen, 2015). The SWAN summary score was used for the present study.

#### ADHD Symptoms (Auxiliary) and Conduct Problems

Teachers completed the Strengths and Difficulties Questionnaire (SDQ, ages 4–10 years; Goodman, 1997) to assess students’ inattention/hyperactivity-impulsivity symptoms and conduct problems scores over the last 6 months on a 3-point Likert scale (*Not True* = 0, *Somewhat True* = 1, and *Certainly True* = 3). The internal consistency of the 25-item questionnaire was generally satisfactory, with a mean a of 0.73 (Goodman, 1997). The internal consistency of the ADHD symptoms and Conduct Problems subscales was also satisfactory, with means α of .88 and .74, respectively (Goodman, 1997). In addition, the reliability of responses was satisfactory (α = .70). Concurrent validity was assessed by examining the association between scores on the SDQ and independently diagnosed disorders according to the Diagnostic Statistical Manual of Mental Disorders – Fourth Edition (DSM-IV; Goodman, 1997). The SDQ’s hyperactivity score was used as an auxiliary variable to students’ missing SWAN scores. SDQ’s scores were strongly correlated with the SWAN’s ADHD symptom scores, *r* = .88, *p* < .001. In addition, the SDQ’s conduct subscale score was used as a covariate in the analyses.

#### Relatedness

Teachers responded to eight items (α = .80) on the Student-Teacher Relationship Quality Scale (STRQ) – Short Form to assess how they get along with individual students (Pianta, 2001). The STRQ uses a 5-point Likert scale ranging from 1 (*definitely does not apply*) to 5 (*definitely applies*). Items were summed, with higher scores reflecting better student-teacher relationships. The same shortened version of the scale was used in another more extensive study with over 2,000 children in Québec (Desrosiers et al., 2012).

#### Autonomy-Support and Structure

Children completed the Structure (four items, α = .76) and autonomy support (eight items, α = .79) subscales of the Student Report of Teacher as Social Context Questionnaire (TASC; Skinner & Belmont, 1993), which consisted of items answered on a 4-point Likert scale, ranging from 1(*not at all true*) to 4 (*very true*). The structure subscale measured student perceptions of teachers’ help/support and adjustment/monitoring. In contrast, the autonomy support scale measured student perceptions of teacher provisions of choice and control in the classroom. Average scores were computed for each of the two scales, where higher scores reflected more autonomy support and structure in the classroom (Ahn et al., 2019).

#### Academic Achievement

Children completed Form A of the letter-word identification and word attack subtests from the Woodcock-Johnson Tests of Achievement-Fourth Edition (WJ-IV ACH; McGrew & Mather, 2014). The WJ-IV ACH is a standardised, individually administered measure designed to assess academic skills across a wide range of content areas. The test is norm-referenced, meaning a student’s performance is compared to that of a nationally representative sample of individuals of the same age or grade. Students were evaluated using subtests that assessed their word-reading skills and phonetic decoding by asking them to read increasingly difficult sight and unfamiliar words. The WJ-IV ACH has good internal consistency (α = .84–.97), test-retest reliability (α = .83–.95), as well as adequate content validity, construct validity, concurrent validity, and clinical validity (McGrew & Mather, 2014; Villareal, 2015). Age-based standardised scores on the letter-word identification and word attack subtests were computed and used as a covariate for children’s academic abilities.

### Analysis Plan

Regression analyses were conducted using *M*plus (Version 8.8; Muthén & Muthén, 2022). The models were performed using Full Information Maximum Likelihood to handle missing data and an estimator (MLR) that corrects for deviations from normality (Geiser, 2012). We also included an auxiliary variable (ADHD symptoms on the SDQ) to improve the missing data estimation (Graham, 2003), mainly to deal with missing data on the SWAN scales.

**Figure 1.**
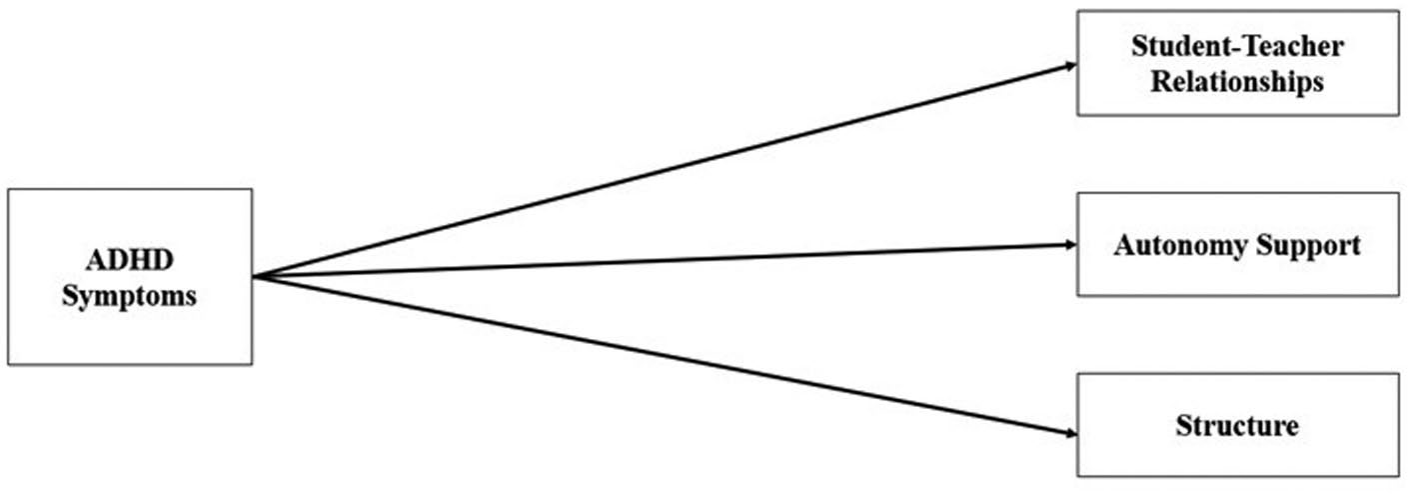
Linear regression model. *Note.* The figure above delineates the linear regression models used to test the three study objectives.

We ran three separate regression models with ADHD symptoms as the independent variable and an NSP (student-teacher relationship quality, autonomy support, or structure) as the dependent variable (see Figure 1). Relevant covariates, including sex, conduct problems, and academic achievement, were also included in the models alongside the predictor variable, ADHD symptoms. The study design comprised subsets of children in the same classroom taught by the same teacher. Due to the non-independence of the observations (i.e., children were nested in classrooms), we used the TYPE=COMPLEX option in *M*plus to correct for the standard errors. When addressing error associated with clustered sample data, McNeish (2023) reported that neither multilevel (i.e., nested) nor single-level modeling approaches are inherently superior, as they rely on differing assumptions and serve different inferential goals.

## Results

### Descriptive Statistics and Correlations

**Figure 2.**
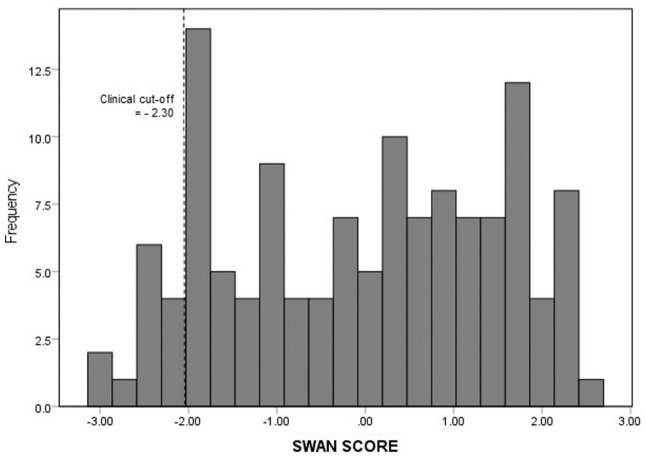
Sample distribution of teacher-reported ADHD symptoms. *Note.* The histogram illustrates the distribution and clinical cut-off (dashed line) score of teacher-reported ADHD symptoms (SWAN Score) on the Strengths and Weaknesses of ADHD-symptoms and Normal Behaviour (SWAN rating scale; Swanson et al., 2012) within this study.

Children were between the ages of 6 to 7 years (*M* = 6.67, *SD* = 0.41), with 51% of the sample indicated as male (see Table 1). In the present sample, child ADHD symptom scores (*M* = −0.17, *SD* = 1.51) were relatively normally distributed and similar to previously reported community-based samples (*M* = −0.57, *SD* = 1.63; Swanson et al., 2012; see Figure 2). Likewise, child inattentive (*M* = −0.06, *SD* = 1.59) and hyperactive-impulsive (*M* = −0.28, *SD* = 1.55) symptom scores were comparable to published community-based levels of inattention (*M* = −0.43, *SD* = 1.76) and hyperactivity-impulsivity (*M* = −0.72, *SD* = 1.65) in similarly aged children. In addition, the proportion of children in the present study sample that were rated as having clinically elevated levels of ADHD symptoms (5.84%) was similar to the rate in an earlier community-sample of children the same age (4.28%; Swanson et al., 2012; see Figure 2). A two-tailed Pearson’s *r* correlation was run for all analysis variables (see Table 2). As predicted, child ADHD symptom scores were negatively correlated with student-teacher relationship scores. However, child ADHD symptom scores were not correlated with autonomy support or structure scores. Regarding the covariates, child ADHD symptoms were correlated with children’s sex, with males (*M* = 0.56, *SD* = 1.18) possessing more ADHD symptoms than female (*M* = −0.97, *SD* = 1.40) students. Likewise, ADHD symptoms were positively correlated with conduct problems and negatively correlated with word-reading ability (Word Reading). However, ADHD symptoms were not correlated with decoding skills (Decoding).

**Table 2.** Correlation Statistics for Analysis Variables.

#	Predictor	1	2	3	4	5	6	7	8	*n* (% missing)	*M* (*SD*)
1	ADHD symptoms									121 (21.9)	−.17 (1.51)
2	STR quality	−.64**								149 (3.9)	3.88 (.57)
3	AS	−.003	.04							153 (1.3)	3.36 (.58)
4	Structure	−.06	.12	.18*						153 (1.3)	2.80 (.68)
5	Sex (0 – female, 1 – male)	.50**	−.44**	−.07	−.01	.42*				154 (0)	0.51 (.50)
6	Conduct problems	.58**	−.62**	−.09	−.03	.61*	.23*			148 (4.5)	1.09 (1.75)
7	Word reading	−.19*	.23**	.01	−.02	−.18*	−.15	−.08		152 (1.9)	102.53 (17.75)
8	Decoding	−.18	.20*	−.05	−.03	−.20*	−.10	−.09	.86**	150 (3.2)	103.35 (15.68)

* *p* < .05. **p < .01.

### ADHD Symptoms and Student-Teacher Relationship Quality

ADHD symptoms were negatively associated with student-teacher relationship quality, β = −.31, *SE* = 0.05, 95% CI [−0.41, −0.20], *p* < .001 (see Table 3). The model explained a significant proportion of variance on the outcome variable, *R*^2^ = .55, *p* < .05. Similarly, children’s sex and conduct problems negatively predicted children’s student-teacher relationship quality, β (Sex) = −.20, *SE* = 0.07, 95% CI [−0.33, −0.07], *p* < .05, β (Conduct Problems) = −.40, *SE* = .04, 95% CI [−0.48, −0.32], *p* < .001. Namely, male students and more conduct problems predicted worse student-teacher relationship scores. However, children’s academic ability was not associated with student-teacher relationship quality, as assessed by their word reading (WJ Letter Word Identification) and decoding skills (WJ Word Attack), β (Word Reading) = −.11, *SE* = 0.10, 95% CI [−0.08, −0.31], *ns*, β (Decoding) = −.01, *SE* = 0.09, 95% CI [−0.19, −0.17], *ns*.

**Table 3.** Linear Regression Predicting Teachers’ Motivational Support Practices.

Regression Analyses	β (*SE*)	95% CI
Student-teacher relationship quality on		
ADHD symptoms	−.31 (0.05)**	[−0.41, −0.20]
Sex	−.20 (0.07)*	[−0.33, −0.07]
Conduct problems	−.40 (0.04)**	[−0.48, −0.32]
Word reading	.11 (0.10)	[−0.08, 0.31]
Decoding	−.01 (0.09)	[−0.19, 0.17]
Autonomy support on		
ADHD symptoms	.18 (0.10)*	[0.02, 0.34]
Sex	−.10 (0.07)	[−0.28, 0.07]
Conduct problems	−.17 (0.07)*	[−0.30, −0.03]
Word reading	.20 (0.16)	[−0.11, 0.51]
Decoding	−.21 (0.14)	[−0.47, 0.06]
Structure on		
ADHD symptoms	−.07 (0.12)	[−0.30, 0.16]
Sex	.02 (0.11)	[−0.19, 0.22]
Conduct problems	.01 (0.13)	[−0.24, 0.25]
Word reading	.03 (0.18)	[−0.33, 0.38]
Decoding	−.10 (0.19)	[−0.43, 0.29]

*Note*. Beta (β) and their associated standard errors (SE) are reported in the first two columns. Confidence intervals (CI) [Lower limit confidence interval (LLCI), upper limit confidence interval (ULCI)] are given at the 95%.

* *p* < .05. **p < .001.

### ADHD Symptoms and Teachers’ Autonomy Support

ADHD symptoms were positively associated with children’s reports of teacher provisions of autonomy support, β = .18, *SE* = 0.10, 95% CI [0.02, 0.34], *p* < .05 (see Table 3). The model accounted for a small, insignificant portion of the variance on student-reported autonomy support, *R*^2^ = .04, *ns*. In addition, conduct problems were negatively associated with children’s perception of autonomy support, β = −.17, *SE* = 0.07, 95% CI [−0.30, −0.03], *p* < .05. However, children’s word reading and decoding skill scores were not associated with children’s perception of autonomy support, β (Word Reading) = .20, *SE* = 0.16, 95% CI [−0.11, 0.51], *ns*, β (Decoding) = −.21, *SE* = 0.14, 95% CI [−0.47 −0.06], *ns*.

### ADHD Symptoms and Teachers’ Structure

ADHD symptoms were not associated with children’s reports of teacher provisions of structure, β = −.07, *SE* = 0.12, 95% CI [−0.30, 0.16], *ns* (see Table 3). The model accounted for a small, insignificant portion of variance on student-reported structure, *R*^2^ = .01, *ns*. Similarly, other covariates, such as conduct problems and reading ability (WJ Letter Word Identification; WJ Word Attack), were not associated with children’s perception of teachers’ structure, β (Conduct Problems) = .01, *SE* = 0.13, 95% CI [−0.24, 0.25], *ns*, β (Word Reading) = .03, *SE* = .18, 95% CI [−0.33, 0.38], *ns*, β (Decoding) = −.10, *SE* = 0.19, 95% CI [−0.43, 0.29], *ns*.

## Discussion

The central goal of this study was to understand the associations between children’s ADHD symptoms and their teachers’ NSPs. We hypothesised that ADHD symptoms would be negatively associated with teachers’ NSPs, whereby more ADHD symptoms would predict worse student-teacher relationships and less autonomy support and structure. This study uniquely evaluated these associations among children in the first grade. We also considered teachers’ experience of the student-teacher relationship and children’s perspectives of their teachers’ autonomy support and structure. Utilising these perspectives enabled us to infer how first grade children feel supported regarding their relatedness, competence, and autonomy.

### ADHD Symptoms and Student-Teacher Relationships

Our first objective sought to examine the association between children’s ADHD symptoms and student-teacher relationship quality in first grade. In line with our prediction, teacher-rated child ADHD symptoms were negatively associated with teacher-rated student-teacher relationship quality. It was also found that sex (i.e., male students) and teacher-rated child conduct problems negatively predicted student-teacher relationship quality. Contrary to our prediction, reading scores, used to measure children’s overall academic competency, were not predictive of student-teacher relationship quality. This predicted relationship may be less relevant among young students, as predictions were based on findings from a study with older students who likely relied on teacher support to navigate more complex academic activities (Jerome et al., 2009).

Furthermore, our findings supported previous research using teacher informants and samples of children with diagnosed ADHD (Prino et al., 2016; Zee et al., 2020; Zendarski et al., 2020). Predominantly, our findings indicated that first grade teachers’ experiences with children’s ADHD behaviours in the classroom contributed to their negative impression of the child as indexed by poor student-teacher relationship quality (Prino et al., 2016; Zendarski et al., 2020). As negative student-teacher relationship quality predicts worse school adjustment (i.e., degree of interest, comfort, engagement, and achievement) among first-grade students (Demirtaş-Zorbaz & Ergene, 2019), likely, first grade teachers’ feelings and beliefs about individual students influence the degree of social support children perceive in the classroom context (Hamre & Pianta, 2001). In other words, teachers’ perception of the student-teacher relationship quality may indicate how related children feel in the classroom (Hamre & Pianta, 2001). Thus, future research should consider teachers’ impressions of young children’s ADHD symptoms and the student-teacher relationship quality to infer children’s relatedness needs in Grade 1 classrooms.

### ADHD Symptoms and Autonomy Support

Our second hypothesis examined whether children’s ADHD symptoms would be negatively associated with teachers’ autonomy support independent of conduct problems and academic ability. We unexpectedly found that ADHD symptoms positively predicted teachers’ autonomy support. In addition, conduct problems were negatively associated with teachers’ autonomy support, while children’s standardised reading scores were not associated with teachers’ autonomy support.

The positive association between ADHD symptoms and autonomy support contradicted Rogers and Tannock’s (2018) study and parenting styles literature. If children with ADHD symptoms experienced classrooms as controlling (Rogers & Tannock, 2018), we expected that ADHD symptoms would negatively associate with autonomy support, which grants greater feelings of autonomy in classroom settings. Parenting styles literature also illustrated that externalising problems, like inattention and hyperactivity/impulsivity, elicited fewer autonomy-granting practices and greater harsh control (Hoeve et al., 2009; Pinquart, 2017). Given the null correlation between ADHD symptoms and autonomy support (see Table 2), random spurious effects may have driven the positive association, rendering our result uninterpretable.

Nevertheless, the negative association between conduct problems and autonomy support coincided with findings from the parenting styles literature suggesting that perceived behavioural challenges might drive the autonomy support children perceive from their teachers (Hoeve et al., 2009; Pinquart, 2017). It may be that disruptive behaviours that interfere with teachers’ classroom proceedings are more strongly related to the degree of autonomy support first-grade children experience from their teachers than ADHD symptoms. Thus, early intervention should initially target Grade 1 students’ disruptive behaviours to prevent losses in autonomy support and associated feelings of classroom autonomy.

### ADHD Symptoms and Teachers’ Structure

If children with ADHD (diagnosed and subclinical symptoms) experience motivational deficits in early elementary, then Self-Determination Theory and self-regulatory deficit models postulate that their feelings of competency should correspond to the amount of structure observed in the classroom setting (Morsink et al., 2022; Rogers & Tannock, 2018; Ryan & Deci, 2020). Thus, our third prediction expected that children’s ADHD symptoms would be negatively associated with children’s perception of teachers’ structure (Jang et al., 2010; Skinner & Belmont, 1993). However, ADHD symptoms did not predict teachers’ structure independent of conduct problems and academic competency. Overall, our findings did not corroborate prior research, including Rogers and Tannock’s (2018) study illustrating that children with ADHD symptoms reported feeling less competent than their peers without ADHD symptoms.

Contrary to our predictions, ADHD symptoms did not negatively relate to teacher provisions of autonomy support and structure. Yet, few studies have exclusively researched teachers’ NSPs among young children with ADHD symptoms aged between 6 and 7 years transitioning into elementary school (Portilla et al., 2014; Zendarski et al., 2020). However, our results may provide critical insight into how autonomous, competent, and related this population feels in response to teachers’ NSPs. Our findings demonstrate that teachers’ observation of children’s ADHD symptoms may not be associated with the amount of autonomy support and structure they provide in first grade. While this suggests that ADHD symptoms may not interfere with teachers’ provision of NSPs in early elementary, teachers’ inadequate fulfilment of children’s psychological needs may manifest in later elementary. In a sample with a wide age range of children (i.e., ages 5–11 years), Rogers and Tannock (2018) found small to moderate associations between ADHD symptoms and children’s feelings of autonomy support and competency. The strength of the associations may be related to the difficulty and complexity of academic material experienced by older elementary children. Thus, there may be a developmental sensitivity of teachers’ NSPs, whereby older children may rely more on teachers’ autonomy support and structure to feel autonomous and competent in school than young children. Consequently, future researchers should consider differences in age and educational contexts when evaluating young children’s perception of teachers’ autonomy support and structure and their associated feelings of autonomy and competency.

### Incidental Findings

Other interesting findings arose from our analyses outside of our main objectives. Firstly, we consistently found a lack of association between teachers’ NSPs and academic competency. Other studies have found that lower academic achievement scores predicted worse teacher-reported student-teacher relationships (Desrosiers et al., 2012; Jerome et al., 2009). Similarly, we anticipated that children who performed better academically might prompt their teachers to give them more choice and control in their learning (Vasquez et al., 2016). In addition, we expected that children’s underachievement might incline teachers to increase student monitoring and provide more instrumental aid during lessons (Luman et al., 2005; Morsink et al., 2021; Reddy et al., 2018). However, given that our sample solely consisted of first-grade students, teacher involvement and structure may play a more prominent role for older students navigating more challenging academic environments and conflicting social interests (Desrosiers et al., 2012; Hamre & Pianta, 2005; Jerome et al., 2009). Likewise, the effect of autonomy support on achievement may be observed in later grades when students, especially adolescents, desire greater autonomy in their learning (Vasquez et al., 2016).

Another curious finding was that our results differed depending on the MSP informant. We assessed student-teacher relationship quality using teachers’ perceptions (STRQ-Short Form; Pianta, 2001) and children’s perceptions of their teachers’ autonomy support and structure (TASC-Short Form; Skinner & Belmont, 1993). Interestingly, disruptive behaviours (i.e., conduct problems) were most pertinent in driving how teachers felt about the student-teacher relationship and how children perceived their teachers’ autonomy support. Thus, it is possible that while ADHD symptoms affected teachers’ impressions of the child, they did not affect children’s experiences of their teachers’ provision of choice and control over their learning. Ultimately, our study elucidates the need to consider age-specific contexts and child informants when assessing teachers’ NSPs.

### Theoretical and Practical Implications

Our study found that ADHD symptoms and conduct problems (and not academic competency) predicted worse student-teacher relationship quality. In addition, conduct problems predicted fewer teacher autonomy support practices. Consequently, it may have been the disruptive behaviours that were associated with ADHD symptoms and conduct problems, which often coloured teachers’ impressions and interactions with specific students (Gatfield & Larmar, 2006; Zendarski et al., 2020). To illustrate, Grade 1 teachers may have believed disruptive children required greater control to complete academic tasks and ensure smooth transitions between school activities. In turn, children exhibiting challenging behaviours may have experienced less autonomy-granting behaviours and fewer positive interactions with teachers in the classroom (Thomassin & Suveg, 2012). This cycle of control closely parallels parental styles literature on harsh control, where parents use verbal or physical punishment and intrusiveness to influence and control children’s behaviours and induce compliance (Hoeve et al., 2009; Pinquart, 2017). Over time, harsh parental control only increases children’s disruptive behaviours and predicts problematic developmental and emotional outcomes (Hoeve et al., 2009; Pinquart, 2017). Thus, children with behavioural challenges transitioning to grade school may be at risk of encountering similar outcomes within the school context (Pianta et al., 1997; Thomassin & Suveg, 2012).

Ultimately, this highlights the need to assess how to support teachers in managing early elementary children who display challenging behaviours that interfere with the classroom, consequently contributing to worse student-teacher relationship quality and controlling instructional practices.

### Limitations and Future Directions

The present study has several limitations. First, previous research has suggested that young children with ADHD symptoms may not be reliable informants of their actual efforts and abilities (Muenks & Miele, 2017; Owens et al., 2007). Young children’s responses regarding their teacher’s teaching style may be influenced by answers they believe are expected or favourable rather than their genuine perceptions (Hoza et al., 2010). Yet, a growing number of studies advocate for the inclusion of children’s voices in research, especially for individuals with ADHD symptoms at risk for motivational deficits (Carlson et al., 2002; Hoza et al., 2010; Morsink et al., 2021). A second limitation of our study was using a cross-sectional design. As a result, our study solely offered insight into the associations between ADHD symptoms and teachers’ NSPs when children were in the spring term of their first-grade year. Lastly, our study possessed some limitations regarding our measures. First, limited child sociodemographic data (including teacher demographics) was collected due to disruptions to our data collection processes caused by the COVID-19 pandemic. Second, a significant number of children (19.5%) were not administered the SWAN (the ADHD symptom measure). However, we used the SDQ’s Hyperactivity score, highly correlated with the SWAN summary score (see Table 2), as an auxiliary variable in the model to reduce bias and improve estimation. Second, we used the short form of the TASC, which examined select dimensions of children’s perception of their teachers’ autonomy support (*choice* and *control*) and structure (*instrumental help* and *monitoring*). Together, these limitations may have contributed to our insignificant finding between ADHD symptoms and teachers’ autonomy support and structure. Future research should consider assessing the stability of these associations using a within-group level, longitudinal, and multi-informant approach and with the full-form TASC to evaluate all dimensions (e.g., student-teacher relationship closeness and conflict) of teachers’ NSPs. Additionally, future research should also consider teachers’ perceived support when evaluating best practices for managing behaviours in the classroom. Together, these may inform future interventions in first grade to raise autonomy support and potentially disrupt the cycle of coercion between children exhibiting challenging classroom behaviours and their teachers.

## Conclusion

We sought to understand the associations between young elementary children’s ADHD symptoms and teachers’ NSPs. Our findings suggest that ADHD symptoms and conduct problems are associated with poor student-teacher relationship quality and less autonomy support. Consequently, these results indicate that children exhibiting behavioural problems may harbour unmet feelings of relatedness and agency in the classroom. Notably, these associations were found among children’s first year transitioning to formal grade school, where academic motivational deficits emerge (Ogg et al., 2016; Rogers et al., 2015). As a result, the present research suggests the need for early intervention to enhance student-teacher relationship quality and increase teachers’ autonomy support to protect against losses in motivational resources vital for energising young children’s engagement in learning (Hamre & Pianta, 2001).
